# Supplementary material for: Application of mini-MLST and whole genome sequencing in low diversity hospital extended-spectrum beta-lactamase producing Klebsiella pneumoniae population
Source: PLoS One. 2019 Aug 13;14(8):e0221187. doi: 10.1371/journal.pone.0221187 (PMC6692064; doi:10.1371/journal.pone.0221187)
Supplement: Attachment — Submitted filename: Response to Reviewers.docx [file pone.0221187.s004.docx]

15.7.2019

Dear Dr. Robinson,

based on your decision letter from July, 3^rd^ 2019 we would like to respectfully resubmit our manuscript newly entitled “Application of mini-MLST and whole genome sequencing in low diversity hospital extended-spectrum beta-lactamases producing *Klebsiella pneumoniae* population “ written by Matej Bezdicek et al., for the PLOS One.

We accepted all the reviewers’ comments and they are discussed below item-by-item. Indicated line numbers are for revised manuscript with accepted changes.

Kind regards,

Martina Lengerova, Ph.D.

Center of Molecular Biology and Gene Therapy

Department of Internal Medicine - Hematology and Oncology

University Hospital Brno

Cernopolni 9

613 00 Brno

Czech Republic

| **Journal Requirements** |  |
| --- | --- |
|  |  |
| Language corrections | Our manuscript was reviewed by a native English-speaker. His name is Matthew Smith at Brno Editing, Proofreading & Translation Services ([www.translationandproofreadingservices.com](http://www.translationandproofreadingservices.com)) and his contact e-mail is matsmithj@yahoo.com. |
| **Editor Comments to Author** |  |
| **Comments to the Author:** |  |
| Have the authors made all data underlying the findings in their manuscript fully available? | **Line 119-121** - Data accession for our project was updated in the manuscript. All raw sequencing data are available under the accession number PRJNA515630 in the BioProject database (direct link was also added to the revised manuscript <https://www.ncbi.nlm.nih.gov/bioproject/PRJNA515630>.) |
| **Reviewers' Comments to Author** |  |
| **Reviewer: 1** |  |
| **Minor comments :** |  |
| Not sure where the sequence data/sequence reads are deposited | **Line 119-121** - Data accession for our project was updated in the manuscript. All raw sequencing data are available under the accession number PRJNA515630 in the BioProject database (direct link was also added to the revised manuscript <https://www.ncbi.nlm.nih.gov/bioproject/PRJNA515630>.) |
| The figure legends are short and uninformative. I know they should not repeat was in the text, but I was having trouble understanding exactly how each figure was put together. Perhaps just a little more information is necessary. One thing that was specifically unclear was whether the dendrogram in Fig 3 was on the basis of allele number similarity, or overall sequence similarity. Similarly, it appears from the Methods that allele numbers were used to create the minimum spanning trees in Fig 4 – which seems odd given that the Results implies it is from actual sequence similarity. Can this be clarified? | **Line 193-199; Line 213-217** - We added short legends to Fig 2 and Fig 3 (formerly Fig 3 and Fig 4). We also clarified allele/sequence similarity in the Methods and Results sections. We transferred Fig 2 to the Table and moved it to the supplemental files (S1 Table). |
| Can the authors give some thought to shortening the Discussion? The main point’s conclusions could possibly be stated more clearly and succinctly e.g. “miniMLST is a cost effective means of ruling out epidemiological linkage, but only complete genome analysis can provide strong evidence in favour of epidemiological linkage”. | **Line 241-341** - We rewrote the discussion to highlight the main conclusions |
| Line 61: should be “bacterial” | **Line 62** - Text corrected |
|  |  |
| **Reviewer: 2** |  |
| **Minor comments :** |  |
| **Line 52** – the word “solution” should likely be “intervention”. | **Line 53** - Text corrected |
| **Line 237-238** – The authors indicate they have generated a new automatic algorithm that acts as a conversion key. Is it possible to utilize this conversion key with the existing MLST schema to generate a conversion list of MSLT to melT groups? This would allow for direct comparison of the large existing studies that use MLST with the use of melT groups and will also clarify the resolution of melT groups. I think this would help clarify the potential role of melT groups among a growing list of molecular epidemiology markers of bacterial diversity (e.g. PFGE, MLST, mini-MLST, cgMLST, WGS). | **Line 259-260** - Once you have designed the mini-MLST scheme the algorithm is applicable to any MLST scheme. To run the algorithm, you need a list of ST including allele variants; FASTA file of all genes used in mini-MLST scheme and mini-MLST primers sequences. Added to the manuscript. |
| **Line 63-65** – The authors define CgMLST and SNV analyses, but the descriptions are confusing. In particular, the authors indicate SNV analysis provides a higher resolution power as compared to cgMLST, but then use SNV as a precursor to CgMLST. It would be good to clarify that both are based upon the detection of variants, but while CgMLST is looking at variants in a specific pre-determined set of genes, true whole genome alignment allows for assessment of variants in all regions that are not masked. | **Line 64-71** - We rewrote the definition of cgMLST and SNV analysis to clarify the differences between those two approaches. |
| **Table 1.-** Can the authors please include the percentage of each subgrouping with the count? This will help the reader understand the proportion of isolates, especially within the source section of the table. | **Table 1, Line 96** - Subgroups percentages were added to the Table 1. |
| **Lines 95-97** – the methods listed for use with mini-MLST differ from the methods used in the previous reference within Diagnostic Microbiology and Infectious Disease (citation 14). Can the authors comment on why the methods were changed from the UltraClean Microbial DNA Isolation Kit to the Chelex based extraction? | As we process a large number of isolates we were looking for a more efficient DNA extraction method. Chelex based extraction costs $ 0.5 per sample and takes less than 30 minutes with minimum hands-on time. UltraClean Microbial DNA Isolation Kit costs $ 5 per sample, takes about 60 minutes and requires more hands-on time. The quality and concentration of DNA extracted with Chelex is suitable for use in PCR methods. |
| **Line 122** – Can the authors indicate how they defined ambiguous positions? Was this based upon a percent threshold for variant detection? | **Line 127-128** - The ambiguous base was added to a position when there were at least two different bases and the less common base represented at least 10 % of bases in the target position. We put a note in the manuscript. |
| **Line 235** – please update the number of ST types as it now appears there are 4,054 different MSLT profiles within the Pasteur MLST database. | **Line 257** - The number of MLST profiles was updated in manuscript on 9/7/2019. |
| **Line 249** – Please update the sentence to say “WGS has become a powerful method” | **Line 271** - Text corrected |
|  |  |
| **Major comments :** |  |
| **Title** – As this manuscript focuses heavily on mini-MLST, please add mini-MLST to the title. | **Title, line 1 -** Title updated to “Application of mini-MLST and whole genome sequencing in low diversity hospital extended-spectrum beta-lactamases producing *Klebsiella pneumoniae* population” |
| **Lines 35-37, 57-59, 318-323** – The authors describe the potential for mini-MLST in real-time epidemiology, but point out the need for mini-MLST to be combined with more discriminatory analyses in outbreak/low genetic diversity settings. Can the authors describe in more detail how this progressive application of mini-MLST and WGS would work in active surveillance? In specific, it would be good to identify how mini-MLST could be used to select isolates for WGS and how mini-MLST would be able to identify outbreaks to focus on. | **Line 242-247** - The following paragraph was added to the discussion.  We are currently using the following protocol in routine practice. ESBLp KPN collected from high-risk departments are prospectively tested with mini-MLST to determine MelT. When the strains’ MelT differ, transmission is unlikely. When we observe an increased incidence of one MelT, we check the potential epidemiological linkages and then we decide if there is a possible outbreak and need of WGS analysis. Meanwhile, early epidemiological measures can be implemented to prevent further spread of infection. |
| **Lines 146-156** – Do the authors intend to submit the WGS sequencing data to a repository such as the European Nucleotide Archive? If so, can the authors include the project ID within the manuscript? | **Line 119-121** - Data accession for our project was updated in the manuscript. All raw sequencing data are available under the accession number PRJNA515630 in the BioProject database (direct link was also added to the revised manuscript <https://www.ncbi.nlm.nih.gov/bioproject/PRJNA515630>.) |
| **Line 215-217** – What was the order of collection for the colonization and infection strains? Were colonization strains always collected before outbreak strains? This may be good information to include in a supplemental table. Additionally, can the authors discuss the number of remaining positions within the genome that are accounted for in calculating the number of SNVs between each pair of isolates? | **Fig 2, Line 193** - Collection dates are listed in Fig 2 and also in S1 Table. In one case the rectum isolate was collected three days before the blood culture isolate and in one case the rectum isolate was collected sixteen days before the blood culture isolate. All other colonization isolates were collected before blood culture isolates.  In total 4,179,387 positions were used in SNV analysis. For core genome SNV analysis only, 2,042,000 positions were analysed. We added S2 and S3 Tables to supplementary files, which show SNV distance matrix for all 46 samples for core genome SNV and core and accessory genome SNV respectively. |
| **Line 271-275** – Can the authors make it more clear if this discussion is referring to the 14 isolates described on lines 146-147, the 24 isolates described from lines 146-150, or does this refer to the entire set of 46 isolates (line 155)? If this is either the 14 or 24 isolates, this represents a small subset of the overall 922 isolates. Can the authors discuss the possibility that this is not a broad enough subsample of the total set of ESBL-KPN isolates to truly evaluate the role of WGS in a low diversity population? | **Line 287-290** - This part of the discussion is referring to all 46 sequenced isolates. We clarified this in the relevant part of the discussion.  Our further project will focus on more detailed analysis of the most predominant MelTs, including sequencing of a larger isolate set, long reads sequencing and also plasmid analysis. |
| **Line 301-310** – I am confused about the application of the premise that “the colonization strain is the cause of the subsequent infection in most cases” to the idea of evaluating a hospital outbreak. In particular, hospital outbreaks are characterized by person-to-person transmission, or are mediated by fomites in the environment. Can the authors describe the potential for misclassification of colonizing and infecting strains if the infecting strain was acquired within the hospital environment? | **Line 314-318** - The number of SNV between colonization/infection pair isolates defines the value of the difference level at which the isolates can still be considered.  We clarified this in the relevant part of the discussion.  In our study (and hospital), the infecting strain is collected from blood culture, urine or other primary sterile material. If the patient has an infection with his own colonizing strain, it is possible to tell only when we also have an isolate from this patient from a previous collection. |
| In a time where there is more focus on acquired resistance genes, and in particular how these acquired genes can change the required antimicrobial treatment choices, how can a technique like mini-MLST be used to help target hospital infection control in the case of an outbreak? | Using knowledge of the local population, its stability and epidemiological linkages along with rapid prospective screening can help implement initial epidemiological measures or find and eliminate a potential outbreak source. As such, the Mini-MLST does not provide information on antibiotic resistance genes. However, information on the resistant genes’ presence in certain MelT acquired by the WGS along with the fact that the hospital population seems to be relatively stable over the long term can be used to predict the resistome of the examined strains. However, to confirm, the strains resistome needs to be examined by specific PCR or WGS. |
| **Figure 2** – This figure is very data dense, but does not display well on a single printed page. Additionally, as the discussion of virulence factors is limited within the manuscript, it may be good to reformat this figure or move it to the supplemental files. | **S1 Table -** We changed the format to Table and move it to the supplemental files |
