# Supplementary material for: Application of mini-MLST and whole genome sequencing in low diversity hospital extended-spectrum beta-lactamase producing Klebsiella pneumoniae population
Source: PLoS One. 2019 Aug 13;14(8):e0221187. doi: 10.1371/journal.pone.0221187 (PMC6692064; doi:10.1371/journal.pone.0221187)
Supplement: Attachment — Submitted filename: Response to Reviewers.docx [file pone.0221187.s005.docx]

30.7.2019

Dear Dr. Robinson,

based on your decision letter from July, 22^nd^ 2019 we would like to respectfully resubmit our manuscript entitled “Application of mini-MLST and whole genome sequencing in low diversity hospital extended-spectrum beta-lactamase producing *Klebsiella pneumoniae* population “ written by Matej Bezdicek et al., for the PLOS One.

We accepted all the Academic Editors’ comments and they are discussed below item-by-item. Indicated line numbers are for revised manuscript with accepted changes.

Kind regards,

Martina Lengerova, Ph.D.

Center of Molecular Biology and Gene Therapy

Department of Internal Medicine - Hematology and Oncology

University Hospital Brno

Cernopolni 9

613 00 Brno

Czech Republic

| **Academic Editor Comments to Author** |  |
| --- | --- |
| **Title** |  |
| Should read "beta-lactamase producing", not plural "beta-lactamases producing" | Line 1 - Title corrected |
|  |  |
| **Abstract** |  |
| Lines 32, 35, 39, referring to the analysis of core and accessory genes as "core/accessory" is ambiguous in the abstract, please state as "core and accessory" as done elsewhere in the text | Lines 32, 35 and 39 - Text corrected |
|  |  |
| **Introduction** |  |
| First 2 sentences are very poorly constructed, please revise | Line 44-49 – Text corrected |
| Line 65, suggested wording "of a pre-determined set of.." | Line 65 – Text corrected |
|  |  |
| **Methods** |  |
| Lines 133, 134 suggested wording of "aligned nucleotide sites" instead of ambiguous term "positions" | Line 133 and 134 – Text corrected |
|  |  |
| **Results** |  |
| Where is Fig 1? I did not see it in the files attached to this paper | Fig 1 is uploaded in the attachment files |
| Line 175 previous wording is better, suggested wording "(including separation of isolate S13 from other isolates)" | Line 175-176 – Text corrected |
| Lines 187 and 190 "average number of different alleles" is more precise language, please revise as appropriate | Lines 187 and 190 – Text corrected |
| Fig3 shows a major topology difference between panels A (core) and B (core and accessory) in the switching of the position of the MelT281, ST23 isolates and MelT132, ST405 isolates. This should be mentioned. | Lines 229-233 – This information was added to the manuscript |
| Line 234 suggested wording "Based on paired sample analysis,..." | Line 239 – Text corrected |
| Lines 237 to 240 wording is unclear. Do the authors mean "Both the highest SNV values were for pair S39/S40 that had time lapses between samples of 47 days. The next highest SNV values were only 3 SNVs for the core and 5 SNVs for the core and accessory, despite time lapses of up to 90 days." Please revise as appropriate. | Lines 242 to 244 – Text corrected |
|  |  |
| **Discussion** |  |
| Line 263 suggested wording "Since then, we have observed six MelTs that account for 82.8% of isolates, from a total of 38 MelTs..." | Line 267-268 – Text corrected |
| Line 276 instead of "big bacterial populations", suggested wording "large populations of bacteria" | Line 280 – Text corrected |
|  |  |
| **Overall** |  |
| Fig2 legend, Same issue as raised by Reviewer1, is the tree based on number of shared alleles or the sequence similarity in the shared alleles? Mostly likely, this legend should include "is based on sharing of 2,251 core genome targets." Please revise as appropriate. | Line 194 – Fig 2 legend revised |
|  |  |
